# The Effects of MELD-Based Liver Allocation on Patient Survival and Waiting List Mortality in a Country with a Low Donation Rate

**DOI:** 10.3390/jcm9061929

**Published:** 2020-06-19

**Authors:** Paul V. Ritschl, Leke Wiering, Tomasz Dziodzio, Maximilian Jara, Jochen Kruppa, Uwe Schoeneberg, Nathanael Raschzok, Frederike Butz, Brigitta Globke, Philippa Seika, Max Maurer, Matthias Biebl, Wenzel Schöning, Moritz Schmelzle, Igor M. Sauer, Frank Tacke, Robert Öllinger, Johann Pratschke

**Affiliations:** 1 Department of Surgery, Campus Charité Mitte, Campus Virchow-Klinikum, Charité—Universitätsmedizin Berlin, Freie Universität Berlin, Humboldt-Universität zu Berlin, Berlin Institute of Health, 10178 Berlin, Germany; paul.ritschl@charite.de (P.V.R.); leke.wiering@charite.de (L.W.); tomasz.dziodzio@charite.de (T.D.); maximilian.jara@charite.de (M.J.); nathanael.raschzok@charite.de (N.R.); frederike.butz@charite.de (F.B.); brigitta.globke@charite.de (B.G.); Philippa.seika@charite.de (P.S.); max-magnus.maurer@charite.de (M.M.); matthias.biebl@charite.de (M.B.); wenzel.schoening@charite.de (W.S.); moritz.schmelzle@charite.de (M.S.); igor.sauer@charite.de (I.M.S.); robert.oellinger@charite.de (R.Ö.); 2 BIH Charité Clinician Scientist Program, Berlin Institute of Health (BIH), Anna-Louisa-Karsch-Str. 2, 10178 Berlin, Germany; 3 Institute of Biometry and Clinical Epidemiology, Charité—Universitätsmedizin Berlin, Freie Universität Berlin, Humboldt-Universität zu Berlin, Berlin Institute of Health, 10178 Berlin, Germany; jochen.kruppa@charite.de (J.K.); uwe.schoeneberg@charite.de (U.S.); 4 Department of Hepatology and Gastroenterology, Campus Charité-Mitte and Campus Virchow-Klinikum, Charité—Universitätsmedizin Berlin, Freie Universität Berlin, Humboldt-Universität zu Berlin, Berlin Institute of Health, 10178 Berlin, Germany; frank.tacke@charite.de

**Keywords:** liver transplantation, allocation policy, donor shortage, organ donation, Germany

## Abstract

The Model for End-Stage Liver Disease (MELD)-based allocation system was implemented in Germany in 2006 in order to reduce waiting list mortality. The purpose of this study was to evaluate post-transplant results and waiting list mortality since the introduction of MELD-based allocation in our center and in Germany. Adult liver transplantation at the Charité—Universitätsmedizin Berlin was assessed retrospectively between 2005 and 2012. In addition, open access data from Eurotransplant (ET) and the German Organ Transplantation Foundation (DSO) were evaluated. In our department, 861 liver transplantations were performed from 2005 to 2012. The mean MELD score calculated with the laboratory values last transmitted to ET before organ offer (labMELD) at time of transplantation increased to 20.1 from 15.8 (Pearson’s R = 0.121, *p* < 0.001, confidence interval (CI) = 0.053–0.187). Simultaneously, the number of transplantations per year decreased from 139 in 2005 to 68 in 2012. In order to overcome this organ shortage the relative number of utilized liver donors in Germany has increased (85% versus 75% in non-German ET countries). Concomitantly, 5-year patient survival decreased from 79.9% in 2005 to 60.3% in 2012 (*p* = 0.048). At the same time, the ratio of waiting list mortality vs. active-listed patients nearly doubled in Germany (Spearman’s rho = 0.903, *p* < 0.001, CI = 0.634–0.977). In low-donation areas, MELD-based liver allocation may require reconsideration and inclusion of prognostic outcome factors.

## 1. Introduction

With the increasing success of modern transplant medicine, the desire for life-saving transplantation has unmasked the lack of appropriate donors [1]. Ongoing demographic changes in both the donor and the recipient pool exacerbate an existing problem faced by clinics and attending physicians: organ allocation. As a consequence, most countries have implemented severity scores (e.g., Model for End-Stage Liver Disease, MELD) in liver allocation, with the aim of guaranteeing help for critically ill recipients and thereby reducing waiting list mortality [2,3]. MELD-based allocation naturally favors more critically-ill transplant candidates, culminating in areas with low rates of organ donation such as Germany [4,5]. Although a high MELD score represents a major risk factor for death while on the waiting list and therefore a need for urgent transplantation [6], it has simultaneously been identified as a major risk factor of post-transplant patient survival [4,7,8,9]. This creates an ethical conflict in terms of the utilization of limited resources with respect to the balance between individual urgency and fair allocation [10].

In Germany, MELD-based liver allocation was implemented on 16 December 2006. The hypothesis of this study was that MELD allocation led to a downward spiral of increasingly ill LT recipients in areas with low donation rates and therefore a deterioration in post-transplant survival outcomes. To our knowledge, this is the first study to evaluate the long-term effects of implementation of MELD-based liver allocation in an area with a low organ donor rate on patient survival and waiting list mortality.

## 2. Patients and Methods

### 2.1. Retrospective Single-Center Analysis

A retrospective analysis of MELD-based liver transplant recipients was performed between 1 January 2005 and 31 December 2012 at the Department of Surgery at Charité—Universitätsmedizin Berlin, a high-volume transplant center in Germany. The allocation system was changed on 16 December 2006; the years 2005 and 2006 were therefore considered to form part of the pre-MELD-allocation phase. Patients with combined transplantations other than combined liver–kidney transplantation, pediatric patients, and patients receiving living donation transplantation were excluded due to the diverging allocation algorithms and therefore disparities in access to liver transplantation. The follow-up period ended on 31 December 2017. The Charité’s Ethics Committee approved the study (number: EA1/369/16).

Independent of medical indication and allocation modus (high urgency, regular MELD-allocation, or rescue allocation), patient survival was analyzed as a primary outcome to evaluate the development of the liver transplant program at our center since the implementation of MELD allocation. Due to the allocation algorithm, two different MELD values were analyzed per patient: the MELD score calculated with the laboratory values last transmitted to ET before organ offer (labMELD) and the labMELD or standard/non-standard exceptional MELD which was applied in allocation (matchMELD). For patients with a high urgency (HU) status, the matchMELD was analyzed as 40 to reflect their prioritization in the allocation system.

### 2.2. Analysis of the Development in the ET Region and Germany

In addition, a secondary data analysis one decade after MELD implementation was performed with open access data from Eurotransplant (ET) and the German Organ Transplantation Foundation (Deutsche Stiftung Organtransplantation, DSO) [11,12,13]. This included epidemiological data pertaining to both donor and recipient characteristics as well as waiting list dynamics (e.g., patients registered per year, number of active patients on waiting list, number of removals from the waitlist and reasons thereof, and survival rates).

### 2.3. Statistical Analysis

Investigated variables are shown as mean ± standard deviation or if not normally distributed as median + first and third quartile. Primary and secondary endpoints were analyzed using appropriate parametric or non-parametric statistical methods based on their scale and distribution. This included the chi-squared test for categorical variables, the Mann–Whitney U test for nonparametric continuous variables, the unpaired *t*-test for normally distributed values, Kaplan–Meier survival analysis, and the log-rank test for survival differences. The Pearson correlation coefficient was computed for testing associations with time. Where applicable, two-tailed tests were performed. Data were tested for normal distribution via visual analysis using histograms. Multivariate statistics was not performed, as too many known and unknown confounders (e.g., inconsistency in the waiting list, decline in organ donors, allocation scandal in Germany in 2012, missing data on organ quality) prohibit meaningful conclusions. All *p*-values from these analyses were considered in an exploratory way due to the study’s retrospective design. A *p*-value below 0.05 was considered as statistically significant. All data analysis was performed using the statistical software IBM SPSS Statistics (IBM Corporation, Armonk, NY, USA, version 25). Shown graphs were created with GraphPad Prism 5.

## 3. Results

### 3.1. Retrospective Single-Center Analysis

#### Patient Demographics

During the period from 1 January 2005 to 31 December 2012, 954 liver transplantations were performed. A cohort of 861 met the inclusion criteria (Figure S1), with a median follow-up of 85 months (interquartile range (IQR) 38.25–121). Detailed demographic data are shown in Table 1.

**Figure 1:**
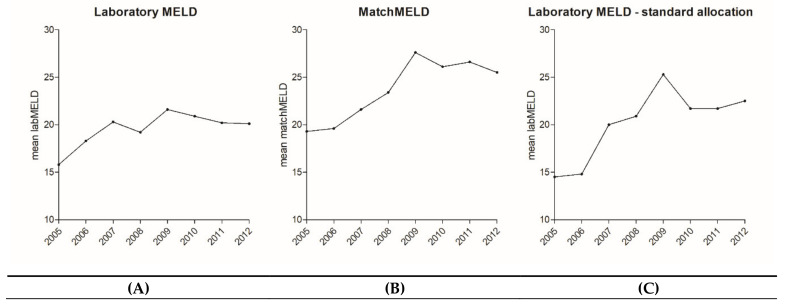
Development of MELD scores after the implementation of MELD-based liver allocation at our transplantation center. Laboratory MELD Score (**A**) as well as matchMELD score (**B**) and sub-analysis of patients without any prioritization (i.e., without patients with standard/non-standard exceptional MELD or high urgency status) (**C**) show a significant increase in the current allocation system after the implementation of MELD-based organ allocation (all *p* < 0.001). This indicates that patients became sicker at the time they received a donor organ over the years. MELD, Model for End-Stage Liver Disease.

On 16 December 2006, MELD-based allocation was implemented in Germany. Since implementation, the mean ET-labMELD at transplantation significantly increased, from 15.8 in 2005 to 20.1 in 2012 (Pearson’s R = 0.121, *p* < 0.001, confidence interval (CI) = 0.053–0.187), largely attributed the fact that patients with high-MELD scores preferentially receive organ offers (Figure 1A).

MatchMELD increased from 2007 (first year of MELD allocation) to 2012 from 21.6 to 25.5, respectively (Pearson’s R = 0.259, *p* < 0.001, CI = 0.0195–0.321) (Figure 1B). In addition, sub-analysis was performed by excluding patients with HU status and standard and non-standard exceptional MELD allocation. Within the subgroup of regularly allocated organs, the ET labMELD increase was more pronounced, rising from 14.5 to 22.5 (Pearson’s R = 0.261, *p* < 0.001, CI = 0.186–0.333) (Figure 1C).

### 3.2. Development of Patient Survival

Five-year patient survival after liver transplantation showed a significant decrease (from 79.9% in 2005 to 60.3% in 2012; log rank *p* = 0.048). Five-year survivors showed significantly lower initial MELD scores (labMELD (*p* = 0.02) as well as matchMELD (*p* = 0.005)) and were less frequently hospitalized prior to transplantation (*p* < 0.001) (Table 2).

**Figure 2:**
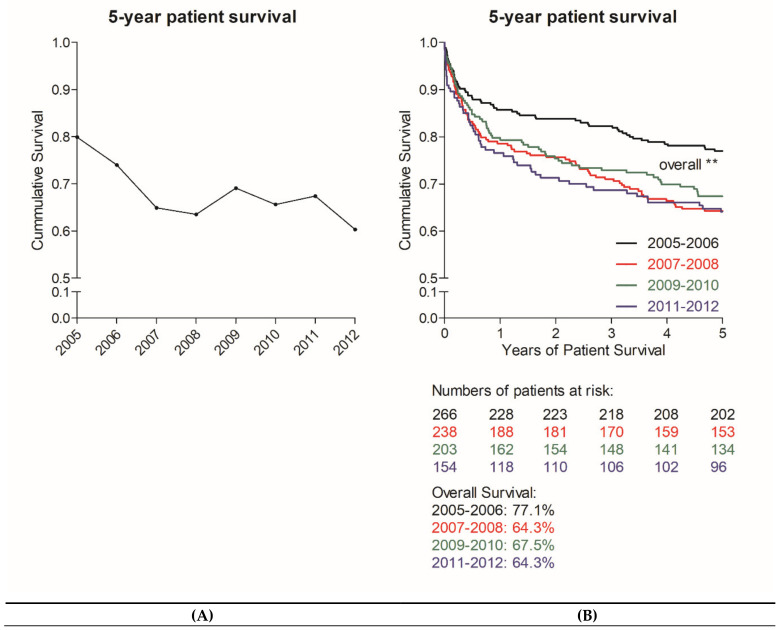
Long-term patient survival after the implementation of MELD-based liver allocation at our transplantation center. In the investigated period we found a significant reduction in long-term patient survival (5-year) after liver transplantation, from 79.9% in 2005 to only 60.3% in 2012 (**A**) (log-rank for all years *p* = 0.048). In comparison to the pre-MELD era (2005–2006) all periods after the implementation of MELD-based organ allocation showed inferior survival (**B**) (log-rank analysis: 2005–2006 vs. 2007–2012, *p* = 0.007; 2005–2006 vs. 2007–2008, *p* = 0.002; 2005–2006 vs. 2009–2010, *p* = 0.023; 2005–2006 vs. 2011–2012, *p* = 0.003). MELD, Model for End-Stage Liver Disease.

Among the eventual non-survivors, patients more frequently received organs from older donors (*p* = 0.036) and organs with a longer cold ischemia time (*p* = 0.018); additionally, they were more likely to receive dialysis prior to transplantation (*p* < 0.001). This descriptive table underlines the fact that at our center the increased mortality originated from a combination of critically ill patients, marginal organs, and therefore detrimental surgical prerequisites. Notably, we found inferior survival rates throughout all time periods during the MELD era compared to those reported between 2005 and 2006 (Figure 2) in Kaplan–Meier analysis. In line with these findings, some existing outcome scores also increased over time, though not all significantly (Figure S2). Therefore, we conclude that the deterioration of recipient survival after implementation of MELD-based liver allocation did not progress over time, but can be attributed to a combination of donor and recipient risk factors.

### 3.3. Analysis of the Development in Germany Compared to the ET Region

#### 3.3.1. MELD Development and Patient Survival

**Figure 3:**
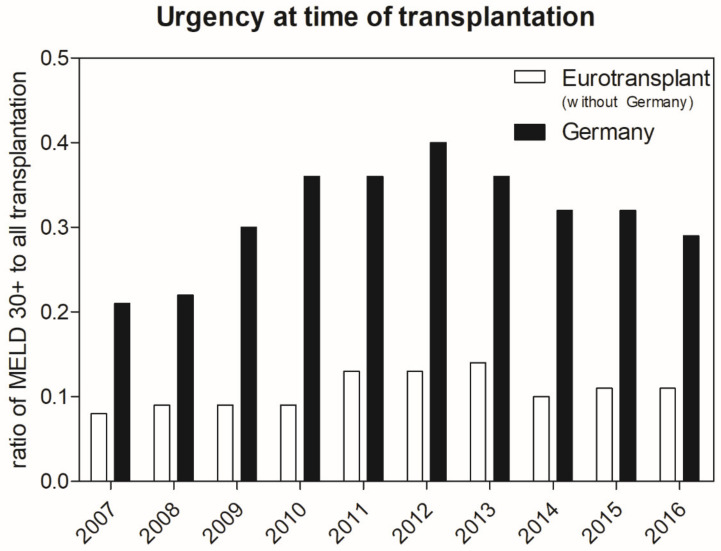
Urgency at time of transplantation in German compared to the Eurotransplant area. Urgency is displayed as the ratio of patients with a high MELD score (MELD ≥ 30). Evidently, liver transplant recipients in Germany have a higher urgency compared to the other partner countries of Eurotransplant (*p* < 0.001). This difference may be caused by several factors, such as different laws concerning organ donation (e.g., opt-out rule in Belgium, Austria, and others) and type of allocation (e.g., center-based allocation). Source: Annual reports of Eurotransplant. MELD, Model for End-Stage Liver Disease.

ET is an international collaborative framework that is responsible for organ allocation in Austria, Belgium, Croatia, Germany, Hungary, Luxembourg, the Netherlands, and Slovenia. Although allocation is fused within this unique organization, country-specific allocation policies (e.g., center-based organ allocation in Austria) and diverging donor numbers are taken into account. When comparing the distribution of recipient groups according to their urgency for transplantation, it becomes obvious that Germany has a higher proportion of high MELD patients compared to the remaining ET collective (chi^2^ for Germany vs. ET without Germany: *p* < 0.001) (Figure 3). As no national dataset is available, we must indirectly infer the nationwide effects of MELD-base liver allocation. After MELD implementation in Germany, the average 5-year patient survival rate in the Eurotransplant region declined from an average rate of 73% in the 5 years prior to MELD implementation in Germany to 69% in the 5 years afterwards (Figure S3). During this period, German transplantation centers performed 59.2% to 67.0% of all liver transplantations in the ET region [13].

#### 3.3.2. Waiting List Development in Germany

**Figure 4:**
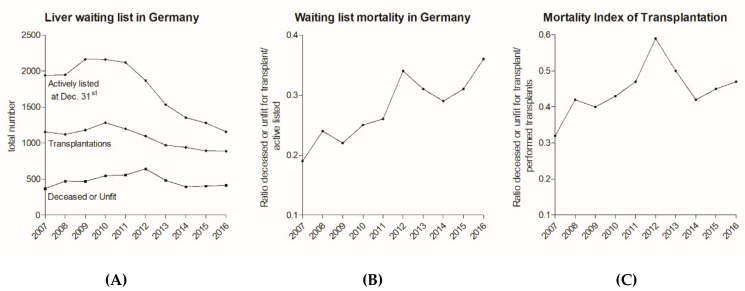
Liver transplant waiting list and waiting list mortality in Germany. While numbers of liver transplantations and actively listed patients on the liver waiting list have drastically declined since 2010, the number of removals from waiting list due to death or “unfit for transplantation” (summarized as waiting list mortality) remained stable in the investigated period (**A**). The ratio of these removals to actively listed patients increased. We found this increase in waiting list mortality to be significant (**B**) (Spearman’s rho = 0.903; *p* < 0.001, CI = 0.634–0.977). The mortality index of transplantation showed a similar development (**C**) (Spearman’s rho = 0.527; *p* = 0.117, CI = −0.153–0.868). Source: Annual Reports of Eurotransplant.

**Figure 5:**
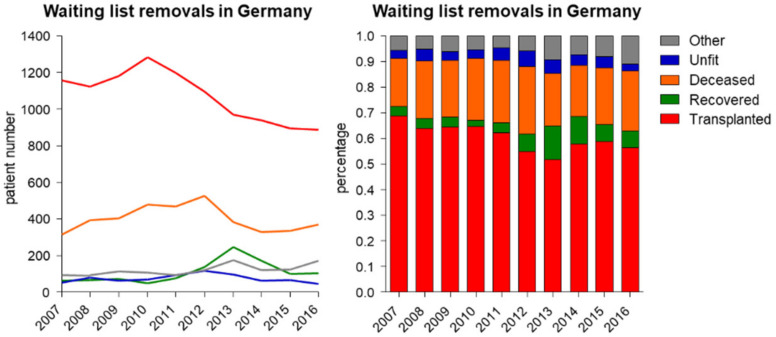
Reasons for removals from the waiting list in Germany. The absolute number of transplantations peaked in 2010, with a consecutive decline (Spearman’s rho = −0.782; *p* = 0.011, CI = −0.945–0.301), but transplantation is still the most frequent reason for delisting. Interestingly, over time the absolute number of recovered patients increased (Spearman’s rho = 0.773; *p* = 0.020, CI = 0.192–0.932), whereas the waiting list mortality (a merge of “deceased” and “unfit”) remained stable over the investigated period (*p* = 0.632 and *p* = 0.838, respectively).

The total number of liver transplantations in Germany peaked in the year 2010 with 1283 before declining to 888 in 2016. Likewise, the number of actively listed patients awaiting liver transplantation declined drastically from 2161 in 2010 to 1157 in 2016. Simultaneously, the absolute number of removals from waiting list due to death or “unfit for transplantation” (summarized as waiting list mortality) remained stable from 2007 until 2016 (Spearman’s rho = −0.067, *p* = 0.855, CI = −0.668–0.587; Figure 4A and Figure 5). However, when these cases are seen in context with the number of actively listed patients a significant increase of this ratio over time is observed (Spearman’s rho = 0.903; *p* < 0.001, CI = 0.634–0.977; Figure 4B). The mortality index of transplantation [14], represented by the ratio of waiting list mortality to performed transplantations, similarly increased, though not significantly (Spearman’s rho = 0.527, *p* = 0.117, CI = −0.153–0.868). The mortality index has the advantage that it is more independent of the waiting list changes and listing regimes (Figure 4C). Surprisingly, the number of patients that were removed from the list due to improved health condition also increased since the introduction of MELD- based liver allocation (Spearman’s rho = 0.733; *p* = 0.020, CI = 0.192–0.932; Figure 5). This underlines that transplantation, apart from exceptional indications, should not be performed below a certain MELD score (e.g., <15), which was achieved via MELD-based allocation in Germany [15].

### 3.4. Donor and Organ Recovery Development in Germany Compared to the ET Region

**Figure 6:**
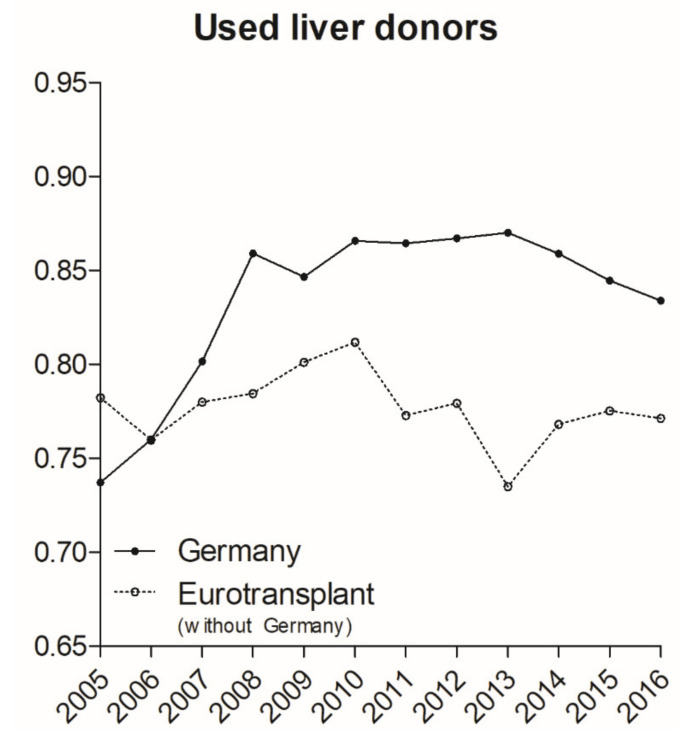
Used liver donors compared to all reported liver donors. While both areas showed a similar utilization of around 75% of all reported liver donors in the years before MELD implementation, the ratio increased in Germany after 2006 to around 85%. Source: Annual Reports of Eurotransplant. MELD, Model for End-Stage Liver Disease.

Since 2010 (15.5/million inhabitants), the number of deceased donors in Germany has steadily decreased. Within the participating countries in the international collaborative framework of ET, Germany has become the nation with the lowest number of deceased donors used per population (9.3/million inhabitants in 2017) [16]. Although this aggravation of this organ scarcity began 2010, the rate of recovered and transplanted liver organs has increased promptly in Germany since MELD implementation. This indicates that the allocation policy change has led to an increased acceptance of marginal organs, independently of short-term changes in organ donors. In contrast, the ratio between possible liver donors and transplantation remained stable in the ET region, and hence a general international trend towards more liberal organ acceptance seems implausible (Figure 6).

## 4. Discussion

We report the first long-term results following the nationwide implementation of MELD-based liver allocation in Germany. The modification of allocation to an urgency-based system has led to significant changes in pre-transplant MELD scores and transplant survival rates and has also affected waitlist characteristics. Following implementation in December 2006, a number of early evaluations of the impact on the German liver transplantation program were published [5,7,17,18]. However, as variations between single years can be influenced by various factors, long-term observations are necessary to evaluate trends.

The allocation of organs according to urgency results in only the sickest patients, as reflected by a high MELD score, receiving a donor organ. To receive organ offers, the clinical condition of moderately sick patients must first deteriorate. The clinical consequences of this trend become particularly evident in areas with low donation rates like Germany. In this study, we were able to confirm the hypothesis that MELD allocation was associated with increasing labMELD as well as matchMELD scores. The effects of transplanting more critically ill patients were also seen in an increase in operating time and increased requirements for blood transfusions during surgery. Generally, recipients in Germany are more likely to have high MELD scores (MELD of ≥30) compared to the rest of the ET region (Figure 4). This effect may partly be explained by higher donor numbers in the other ET countries, but also by the fact that not all countries in ET adhere to “pure” MELD-based allocation (e.g., center-based allocation in Austria).

The prioritization of critically ill patients is a major problem in Germany because, as demonstrated, it affects post-transplant outcome. We report a strong decrease in 5-year patient survival from the preMELD to the MELD era. This is consistent with early analyses, which showed a reduced 3-month survival and a low 1-year survival rate of only 75.8% [7,18]. Although several other countries have implemented MELD-based liver allocation, this trend has not been reported in countries such as the United States, Switzerland, or Brazil [2,19,20]. However, Eurotransplant region patient survival also decreased after 2006. This may be explained by the fact that Germany contributed between 59.2% and 67.0% of all liver transplantations during the period analyzed (Figure S3).

One main difference between organ transplantation in Germany and most other countries is the extremely low rate of organ donation. This decline was first evident in 2011, with a culmination in 2017 when the rate of organ donation dropped below 10 donors per million inhabitants for the first time (ET annual report 2017). As we have demonstrated, the low number of available donor organs results in an increased acceptance of donor livers (including marginal organs). However, while exacerbated by low donation rates, this rise in organ utilization did not occur in correlation with donor decline but rather directly after MELD implementation while donation rates were still relatively high. Organ utilization increased from 73.7% in 2005 (813/1103) before MELD implementation to 87% (750/862) in 2013. It is apparent that the number of critically ill high-MELD patients increasingly compels transplantation professionals to accept marginal organs, resulting in a further compromised transplantation outcome. Non-acceptance in such an unfavorable situation may result in the immediate death of the recipient. In contrast to other countries, donation after cardiac death is not permitted in Germany, even though donor numbers could be considerably augmented [1].

Nonetheless, donation rates in Germany have never been high compared to other countries within Eurotransplant and the decline in recent years is not an entirely new development but rather compounds this existing problem. Hence, the authors believe that the decrease of donation rate is not sufficient to explain changes in survival rates and increasing MELD scores in recent years. These changes occurred shortly after the implementation of MELD allocation and then remained stable independent of changes in donor availability (Figure 2B and Figure 6). Therefore, we speculate that MELD-based allocation caused these changes, which were further compounded by the low organ donation rate.

The aim of MELD-based allocation was not to optimize recipient survival but to reduce waiting list mortality. The reduction of waiting list mortality was particularly pronounced in patients with a high MELD score. This purpose was achieved in North America as well as several European countries [2,21,22]. However, only one study has analyzed changes in waiting list mortality in the German transplantation system to date. Benckert et al. reported a decrease in mortality on the waiting list during the first two years after MELD-based allocation in a single center study [17], but long-term data are missing.

To evaluate waiting list mortality and evaluate the long-term consequences, a closer look at the waiting list epidemiology is necessary. We were able to demonstrate that the characteristics of the liver waiting list have changed profoundly over the last decade in Germany (Figure 4A). The number of transplantations and actively listed patients has consistently declined since 2010. A shrinking waiting list indicates changes in the listing algorithms of transplant centers, probably due to the allocation policy change. Low-MELD patient listing is often postponed to later time points with increased MELD score and consecutive realistic chances for transplantation.

The definition and comparison of waiting list mortality over time remains controversial. Most studies define waiting list mortality as death per 100 patient years on the waiting list, as proposed by the Scientific Registry of Transplant Recipients [23]. This seems reasonable but requires stable listing criteria, which is not the case in Germany where listing policies have been changed due to MELD allocation and organ shortage. Hence, mortality per patient years at risk seems an unsuitable parameter for Germany, as it will be erroneously high due to the comparatively fewer “healthy” patients listed. In our opinion, evaluation of waiting list mortality in Germany should be independent of the waiting list. Therefore, the absolute number of mortalities in fixed regions (e.g., Germany) or the ratio of mortality per transplantation (mortality index) seem favorable. In the former case the total numbers of deceased patients were found to be stable over the last decade (Figure 4A). While low-MELD patients are not listed, critically ill patients with a high risk of deceasing while on the waiting list—as represented by a high MELD score—are more likely to be listed as they urgently need transplantation. Consequently, the absolute numbers in a fixed region would be independent of listing policies. In contrast, relative mortality ratios (e.g., per transplantations performed or listed patients) demonstrated significant increases in this study (Figure 4B,C), most likely driven by the fact that fewer transplantations were performed/fewer patients were on the waiting list. Finally, interpretation of waiting list mortality in the context of variable listing policies, donation rates, and waiting list size remains difficult and debatable. However, according to our data there is no evidence that implementation of MELD-based allocation was able to sustainably reduce waiting list mortality in Germany.

Due to Its Retrospective Character, This Study Has Several Limitations

Firstly, we report a single-center experience and therefore our results cannot be generalized to every transplantation center in Germany. Unfortunately, no German transplantation registry is available as yet to answer these questions sufficiently. Furthermore, international organ donation and transplantation programs vary substantially in many aspects (acceptance of deceased donation including donation after cardiac arrest, allocation rules, rate of living donor transplantation, etc.). Hence, while conclusions may not be transferred directly, the lessons learned from this experience after MELD implementation in Germany can be of great value for organ transplantation regulations internationally.

Secondly, since MELD implementation in 2006, many factors have influenced liver transplantation rates in Germany. Besides changes in the allocation algorithm, there was considerable variation in the number of organ donors between 2009 and 2016, with numbers ranging from 1200 to 2200 donors. This followed a liver allocation scandal which was uncovered in 2012 and had a considerable detrimental effect on the public perception of transplantation. The listing criteria of many centers were modified as a consequence to adapt to all these dramatic changes. Hence, a relationship between the reduction in patient survival in our center and the effect on waiting list mortality with the implementation of MELD-based liver allocation remains inconclusive and most likely is a result of the interaction between multiple contributing factors.

## 5. Conclusions

After the implementation of MELD-based liver allocation and in the context of increasing organ shortage, patient survival after transplantation decreased both in our center and in the overall ET region. Furthermore, a significant reduction of waiting list mortality could not be demonstrated with the available datasets. Therefore, a re-evaluation of liver allocation in Germany appears reasonable, and prognostic outcome factors should be considered for allocation.

## Figures and Tables

**Table 1:** Patient and donor characteristics.

	All	2005	2006	2007	2008	2009	2010	2011	2012	Correlation	
	*n* = 861	*n* = 139	*n* = 127	*n* = 134	*n* = 104	*n* = 110	*n* = 93	*n* = 86	*n* = 68	Method *p*-Value	
Age at LT *	53.1 (±10.3)	49.9 (±11.0)	51.1 (±11.1)	55.0 (±7.4)	55.9 (±9.8)	53.2 (±10.6)	53.6 (±10.3)	53.7 (±10.1)	54.2 (±10.4)	R 0.108	**0.001**
Male sex ***	560 (65.0%)	83 (59.7%)	83 (65.4%)	87 (64.9%)	72 (69.2%)	67 (60.9%)	57 (61.3%)	64 (74.4%)	47 (69.1%)	Rho	0.149
BMI (kg/m^2^) *	26.4 (±4.9)	25.2 (±4.2)	25.5 (±4.9)	26.9 (±4.6)	25.8 (±4.7)	27.3 (±4.7)	27.0 (±5.9)	27.0 (±5.0)	27.0 (±5.0)	R 0.133	**<0.001**
Indication ***											
Cirrhosis	347 (40.3%)	47 (33.8%)	50 (39.4%)	63 (47.0%)	41 (39.4%)	46 (41.8%)	42 (45.2%)	33 (38.4%)	25 (36.8%)		
Virus-related	77 (8.9%)	16 (11.5%)	18 (14.2%)	14 (10.4%)	7 (6.5%)	6 (5.4%)	8 (8.6%)	3 (3.5%)	5 (7.6%)		
Alcoholism	205 (23.8%)	22 (15.8%)	27 (21.3%)	41 (31.6%)	30 (28.8%)	23 (20.9%)	26 (28.0%)	23 (26.7%)	13 (19.1%)		
Combined viral + alcoholic	9 (1.0%)	2 (1.4%)	0	1 (0.7%)	1 (1.0%)	2 (1.8%)	1 (1.1%)	0	2 (2.9%)		
Other	71 (6.4%)	7 (5.0%)	5 (3.9%)	7 (5.2%)	3 (2.9%)	15 (13.6%)	7 (7.5%)	7 (8.1%)	5 (7.6%)		
Liver tumor	241 (28.0%)	28 (20.1%)	30 (23.6%)	34 (25.4%)	38 (36.5%)	36 (32.7%)	28 (30.1%)	26 (30.2%)	21 (30.9%)		
Retransplantation	120 (13.9%)	30 (21.6%)	19 (15.0%)	18 (13.4%)	11 (10.6%)	11 (10.0%)	13 (14.0%)	10 (11.6%)	8 (11.8%)		
Primary non-function	28 (3.3%)	9 (6.5%)	4 (3.1%)	2 (1.5%)	3 (2.9%)	3 (2.7%)	4 (4.3%)	3 (3.5%)	0		
Hepatic artery thrombosis	29 (3.4%)	11 (7.9%)	7 (5.5%)	3 (2.2%)	1 (1.0%)	1 (0.9%)	3 (3.2%)	1 (1.2%)	2 (2.9%)		
ITBL	26 (2.4%)	4 (2.9%)	0	7 (5.2%)	3 (2.9%)	3 (2.7%)	2 (2.2%)	1 (1.2%)	1 (1.5%)		
Recurrent disease	16 (1.9%)	2 (1.4%)	4 (3.1%)	2 (1.5%)	1 (1.0%)	0	1 (1.1%)	4 (4.7%)	2 (2.9%)		
Rejection	9 (1.0%)	2 (1.4%)	1 (0.8%)	0	2 (1.9%)	2 (1.8%)	1 (1.1%)	0	1 (1.5%)		
Other	17 (1.4%)	2 (1.4%)	3 (2.4%)	4 (3.0%)	1 (1.0%)	2 (1.8%)	2 (2.2%)	1 (1.2%)	2 (2.9%)		
Cholestatic disease	65 (7.5%)	19 (13.7%)	9 (7.1%)	10 (7.5%)	7 (6.7%)	5 (4.5%)	6 (6.5%)	6 (7.0%)	3 (4.4%)		
Acute liver failure	34 (3.9%)	3 (2.2%)	9 (7.1%)	6 (4.5%)	2 (1.9%)	7 (6.4%)	1 (1.1%)	5 (5.8%)	1 (1.5%)		
Other	54 (6.3%)	12 (8.6%)	7 (5.5%)	3 (2.2%)	5 (4.8%)	5 (4.5%)	3 (3.2%)	6 (7.0%)	10 (14.7%)		
**Listing details**											
Waiting time in days **	106 (17.5–327.5)	182 (56–471)	141 (24–441)	61 (12.75–275)	102 (29–228.5)	77.5 (8.75–242)	96 (20–325.5)	111 (19–324.25)	47 (11.5–227)	Rho −0.127	**<0.001**
Allocation labMELD *	19.5 (±10.2)	15.8 (±6.7)	18.3 (±9.1)	20.3 (±10.3)	19.2 (±10.3)	21.6 (±11.8)	20.9 (±10.5)	20.2 (±11.4)	20.1 (±10.7)	R 0.121	**<0.001**
matchMELD *	23.3 (±10.6)	19.3 (±11.0)	19.6 (±10.8)	21.6 (±10.5)	23.4 (±9.7)	27.6 (±9.2)	26.1 (±9.6)	26.6 (±10.1)	25.5 (±9.5)	R 0.259	**<0.001**
labMELD prior OLT *	19.7 (±10.2)	18.8 (±9.0)	19.2 (±9.6)	19.9 (±10.0)	19.0 (±10.0)	21.4 (±11.6)	20.1 (±9.8)	20.0 (±11.1)	19.8 (±10.6)	R	0.248
High urgency status ***	133 (12.1%)	22 (15.8%)	20 (15.7%)	9 (6.7%)	7 (6.7%)	13 (11.8%)	9 (9.7%)	8 (9.3%)	6 (8.8%)	Rho −0.069	**0.043**
Retransplantation ***	120 (13.9%)	30 (21.6%)	19 (15.0%)	18 (13.4%)	11 (10.6%)	11 (10.0%)	13 (14.0%)	10 (11.6%)	8 (11.8%)	Rho −0.079	**0.02**
First retransplant	103(12.0%)	26 (18.7%)	15 (11.8%)	16 (11.9%)	11 (10.6%)	11 (10.0%)	11 (11.8%)	7 (8.1%)	6 (8.8%)		
Second retransplant	16 (1.9%)	4 (2.9%)	4 (3.1%)	2 (1.5%)			2 (2.2%)	2 (2.3%)	2 (2.9%)		
Third retransplant	1 (0.1%)							1 (1.2%)			
**Graft and surgical parameters**											
CIT (min) *	578.1 (±166.3)	585.1 (±142.1)	554.5 (±173.8)	601.2 (±164.6)	626.8 (±172.7)	574.5 (±154.1)	556.9 (±170.6)	546.3 (±195.7)	562.6 (±147.9)	R	0.122
Operating time (min) *	316 (±86.1)	330 (±94.3)	316.5 (±73.2)	310.5 (±84.4)	332.9 (±82.7)	337.7 (±80.2)	337.6 (±87.1)	336.5 (±91.3)	365.3 (±90.2)	R 0.119	**<0.001**
WIT (min) *	46.4 (±12.1)	45.7 (±10.7)	42.4 (±10.0)	41.0 (±9.2)	45.0 (±13.3)	50.2 (±12.5)	48.7 (±12.8)	50.5 (±10.7)	50.1 (±14.5)	R 0.218	**<0.001**
Erythrocyte concentrate **	6 (3–10)	4 (2–6)	4 (2–6)	6 (3–10)	7 (4–13)	6 (3–10)	6 (4–11.75)	6.5 (3–12)	9.5 (6–14)	Rho 0.272	**<0.001**
FFP **	18 (13–28)	12 (8–17)	13 (10–20)	19 (13–27)	22 (16–35)	21 (16–30)	22.5 (15–32)	20 (14–28.5)	22 (15.25–38.75)	Rho 0.351	**<0.001**
Post-transplant ICU stay **	9 (6–19)	8 (6–14)	9 (6–20)	7 (4.75–16.5)	9 (6–15)	9 (5–22)	11 (6–23)	10.5 (6–26.5)	13 (7–22.75)	Rho 0.10	**0.003**
Post-transplant hospital stay **	30 (22–52.5)	30 (25–44)	28 (22–49)	27.5 (22–54.25)	29.5 (21–49.75)	36 (25–56.25)	35 (21.5–50)	33.5 (21.75–56)	33 (18.25–60.5)	Rho	0.336
**Donor characteristics**											
Age *	52.6 (±17.1)	50.1 (±16.7)	50.1 (±17.9)	51.9 (±18.1)	52.0 (±16.5)	56.5 (±15.0)	51.4 (±18.3)	55.0 (±16.0)	57.2 (±16.6)	R 0.118	**0.001**
Male sex ***	427 (49.6%)	62 (44.6%)	67 (52.8%)	67 (50.0%)	49 (47.1%)	53 (48.2%)	50 (53.8%)	44 (51.2%)	35 (51.5%)	Rho	0.39
BMI (kg/m^2^) *	26.0 (±4.5)	25.7 (±4.6)	25.6 (±4.3)	26.3 (±4.6)	26.3 (±4.7)	26.5 (±3.6)	25.5 (±4.2)	26.5 (±5.9)	25.4 (±3.5)	R	0.578
ICU stay **	3 (2–7)	3 (1.5–8)	4 (2–6.25)	3 (1–7)	4 (2–8)	3 (2–5)	3 (1–7.5)	4 (2–9)	2.5 (1–5.5)	Rho	0.667
Reanimation ***	153 (17.8%)	20 (14.4%)	20 (15.7%)	15 (11.2%)	27 (26.0%)	28 (25.5%)	19 (20.4%)	13 (15.1%)	11 (16.2%)	Rho	0.167
Cause of death ***											
SAH	433 (50.3%)	80 (57.6%)	62(48.8%)	65 (48.5%)	52 (50.0%)	60 (54.5%)	38 (40.9%)	39 (45.3%)	37 (54.4%)		
TBI	132 (15.3%)	18 (12.9%)	24 (18.9%)	27 (20.1%)	12 (11.5%)	13 (11.8%)	15 (16.1%)	15 (17.4%)	8 (11.8%)		
Hypoxia	114 (13.2%)	18 (12.9%)	14 (11.0%)	13 (9.7%)	21 (20.2%)	13 (11.8%)	17 (18.3%)	11 (12.8%)	7 (10.3%)		
CVA	91 (10.6%)	11 (7.9%)	13 (10.2%)	13 (9.7%)	10 (9.6%)	14 (12.7%)	9 (9.7%)	13 (15.1%)	8 (11.8%)		
ICH	50 (5.8%)	7 (5.0%)	9 (6.7%)	8 (6.3%)	4 (3.8%)	5 (4.5%)	8 (8.6%)	4 (4.8%)	5 (7.4%)		
Other	41 (4.7%)	5 (3.6%)	6 (4.7%)	7 (5.2%)	5 (4.8%)	5 (4.5%)	6 (6.5%)	4 (4.8%)	3 (4.4%)		

* Mean (±standard deviation); ** Mean (interquartile range); *** count (percentage). BMI, body mass index; CIT, cold ischemia time; CVA, cerebrovascular accident; ET, Eurotransplant; FFP, fresh frozen plasma; ICH, intracranial hemorrhage; ICU, intensive care unit; ITBL, Ischemic type biliary lesions; LT, liver transplantation; allocation labMELD, MELD score calculated with the laboratory values last transmitted to ET before organ offer; matchMELD, labMELD or standard/non-standard exceptional MELD which was applied in allocation; labMELD prior OLT, MELD score calculated with the laboratory values directly prior transplantation; MELD, Model for End-Stage Liver Disease; SAH, subarachnoid hemorrhage; TBI, traumatic brain injury; WIT, warm ischemia time.

**Table 2:** Characteristics of 5-year survivors and non-survivors.

	5-Year Survivors	5-Year Non-Survivors	*p*-Value
	*n* = 594	*n* = 267	
Age at LT *	52.7 (±10.4)	54.1 (±9.9)	0.063
Male sex ***	365 (61.4%)	195 (73.0%)	**0.001**
BMI (kg/m^2^) *	26.5 (±4.9)	26.2 (±5.0)	0.399
Indication ***			**<0.001**
Liver tumor	154 (25.9%)	87 (32.6%)	
Cirrhosis	263 (44.3%)	84 (31.5%)	
Retransplantation	63 (10.6%)	57 (21.3%)	
Acute liver failure	26 (4.4%)	8 (3.0%)	
Other	88 (14.8%)	31 (11.6%)	
Listing Details			
labMELD *	18.8 (±9.8)	21.0 (±10.9)	**0.02**
matchMELD *	22.6 (±10.3)	25.0 (±11.0)	**0.005**
High urgency status ***	64 (10.8%)	30 (11.2%)	0.841
Place ***			
Home	438 (73.7%)	155 (58.1%)	**<0.001**
Regular ward	60 (10.1%)	46 (17.2%)	
ICU	96 (16.2%)	66 (24.7%)	
Dialysis prior to transplantation ***	64 (10.8%)	67 (25.1%)	**<0.001**
Graft and surgical parameters			
Cold ischemia time *	569.2 (±167.7)	598.1 (±161.8)	**0.018**
Erythrocyte concentrate **	5 (2–8)	7 (4–12)	**<0.001**
FFP **	17 (12–25)	20 (15–31)	**<0.001**
Donor Characteristics			
Age *	51.8 (±17.3)	54.4 (±16.5)	**0.036**
BMI (kg/m^2^) *	26.0 (±4.6)	26.0 (±4.2)	0.874
Risk Scores			
D-MELD *	956 (±574)	1099 (±624)	**<0.001**
Donor risk index *	2.4 (±0.5)	2.5 (±0.5)	**0.008**
Balance of risk score *	8.0 (±5.5)	10.5 (±6.7)	**<0.001**

* Mean (±standard deviation), ** Mean (interquartile range), *** count (percentage). BMI, body mass index; D-MELD, product of donor age and preoperative MELD; FFP, fresh frozen plasma; LT, liver transplantation; ICU, intensive care unit; MELD, Model for End-Stage Liver Disease.

## Supplementary Materials

The following are available online at https://www.mdpi.com/2077-0383/9/6/1929/s1, Figure S1: Patients included in the study, Figure S2. Development of established risk scores for liver transplantation after the implementation of MELD-based liver allocation, Figure S3. Development of 5-year patient survival from 2002 to 2012 in the whole Eurotransplant area.

## Abbreviations

BMI	body mass index
CI	confidence interval
CIT	cold ischemia time
CVA	cerebrovascular accident
DSO	German Organ Transplantation Foundation
ET	Eurotransplant
FFP	fresh frozen plasma
HU	high urgency
ICH	intracranial hemorrhage
ICU	intensive care unit
IQR	interquartile range
ITBL	ischemic type biliary lesions
LT	liver transplantation
MELD	Model for End-Stage Liver Disease
SAH	subarachnoid hemorrhage
TBI	traumatic brain injury
WIT	warm ischemia time

## References

[1] Schulte K., Borzikowsky C., Rahmel A., Kolibay F., Polze N., Frankel P., Mikle S., Alders B., Kunzendorf U., Feldkamp T. (2018). Decline in Organ Donation in Germany. Dtsch. Arztebl. Int..

[2] Dutkowski P., Oberkofler C.E., Bechir M., Mullhaupt B., Geier A., Raptis D.A., Clavien P.A. (2011). The model for end-stage liver disease allocation system for liver transplantation saves lives, but increases morbidity and cost: A prospective outcome analysis. Liver Transpl..

[3] Schilsky M.L., Moini M. (2016). Advances in liver transplantation allocation systems. World J. Gastroenterol..

[4] Schlitt H.J., Loss M., Scherer M.N., Becker T., Jauch K.W., Nashan B., Schmidt H., Settmacher U., Rogiers X., Neuhaus P. (2011). Current developments in liver transplantation in Germany: MELD-based organ allocation and incentives for transplant centres. Z. Gastroenterol..

[5] Tacke F., Kroy D.C., Barreiros A.P., Neumann U.P. (2016). Liver transplantation in Germany. Liver Transpl..

[6] Wiesner R., Edwards E., Freeman R., Harper A., Kim R., Kamath P., Kremers W., Lake J., Howard T., Merion R.M. (2003). Model for end-stage liver disease (MELD) and allocation of donor livers. Gastroenterology.

[7] Weismuller T.J., Fikatas P., Schmidt J., Barreiros A.P., Otto G., Beckebaum S., Paul A., Scherer M.N., Schmidt H.H., Schlitt H.J. (2011). Multicentric evaluation of model for end-stage liver disease-based allocation and survival after liver transplantation in Germany—limitations of the *‘*sickest first’-concept. Transpl. Int..

[8] Rana A., Hardy M.A., Halazun K.J., Woodland D.C., Ratner L.E., Samstein B., Guarrera J.V., Brown R.S., Emond J.C. (2008). Survival outcomes following liver transplantation (SOFT) score: A novel method to predict patient survival following liver transplantation. Am. J. Transpl..

[9] Cywinski J.B., Mascha E.J., You J., Sessler D.I., Kapural L., Argalious M., Parker B.M. (2011). Pre-transplant MELD and sodium MELD scores are poor predictors of graft failure and mortality after liver transplantation. Hepatol. Int..

[10] Schaubel D.E., Guidinger M.K., Biggins S.W., Kalbfleisch J.D., Pomfret E.A., Sharma P., Merion R.M. (2009). Survival benefit-based deceased-donor liver allocation. Am. J. Transpl..

[11] Eurotransplant Statistics Report Library. http://statistics.eurotransplant.org/.

[12] Tätigkeitsberichte und Qualitätsberichte. https://www.dso.de/servicecenter/krankenhaeuser/transplantationszentren.html.

[13] Annual Reports. https://www.eurotransplant.org/cms/index.php?page=annual_reports.

[14] Peschel G., Kraft I.C., Scherer M., Sinner B., Huber K., Müller-Schilling M., Weigand K. (2018). Wartelistenmortalität bei der Lebertransplantation—the dark side of liver transplantation. Z. Gastroenterol..

[15] Linecker M., Krones T., Berg T., Niemann C.U., Steadman R.H., Dutkowski P., Clavien P.A., Busuttil R.W., Truog R.D., Petrowsky H. (2018). Potentially inappropriate liver transplantation in the era of the “sickest first” policy—A search for the upper limits. J. Hepatol..

[16] Branger P., Samuel U. (2017). Eurotransplant International Foundation.

[17] Benckert C., Quante M., Thelen A., Bartels M., Laudi S., Berg T., Kaisers U., Jonas S. (2011). Impact of the MELD allocation after its implementation in liver transplantation. Scand. J. Gastroenterol..

[18] Weismuller T.J., Negm A., Becker T., Barg-Hock H., Klempnauer J., Manns M.P., Strassburg C.P. (2009). The introduction of MELD-based organ allocation impacts 3-month survival after liver transplantation by influencing pretransplant patient characteristics. Transpl. Int..

[19] Agopian V.G., Petrowsky H., Kaldas F.M., Zarrinpar A., Farmer D.G., Yersiz H., Holt C., Harlander-Locke M., Hong J.C., Rana A.R. (2013). The evolution of liver transplantation during 3 decades: Analysis of 5347 consecutive liver transplants at a single center. Ann. Surg..

[20] Palmiero H.O., Kajikawa P., Boin I.F., Coria S., Pereira L.A. (2010). Liver recipient survival rate before and after model for end-stage liver disease implementation and use of donor risk index. Transpl. Proc..

[21] Freeman R.B., Wiesner R.H., Edwards E., Harper A., Merion R., Wolfe R. (2004). United Network for Organ Sharing Organ Procurement and Transplantation Network Liver and Transplantation Committee. Results of the first year of the new liver allocation plan. Liver Transpl..

[22] De la Mata M., Cuende N., Huet J., Bernardos A., Ferron J.A., Santoyo J., Pascasio J.M., Rodrigo J., Solorzano G., Martin-Vivaldi R. (2006). Model for end-stage liver disease score-based allocation of donors for liver transplantation: A spanish multicenter experience. Transplantation.

[23] Waitlist Mortality Rate. https://www.srtr.org/about-the-data/guide-to-key-transplant-program-metrics/txguidearticles/waitlist-mortality-rate/.

