# Supplementary material for: The Effects of MELD-Based Liver Allocation on Patient Survival and Waiting List Mortality in a Country with a Low Donation Rate
Source: J Clin Med. 2020 Jun 19;9(6):1929. doi: 10.3390/jcm9061929 (PMC7356806; doi:10.3390/jcm9061929)
Supplement: Supplementary file 1 [file jcm-09-01929-s001.pdf]

Supplementary materials

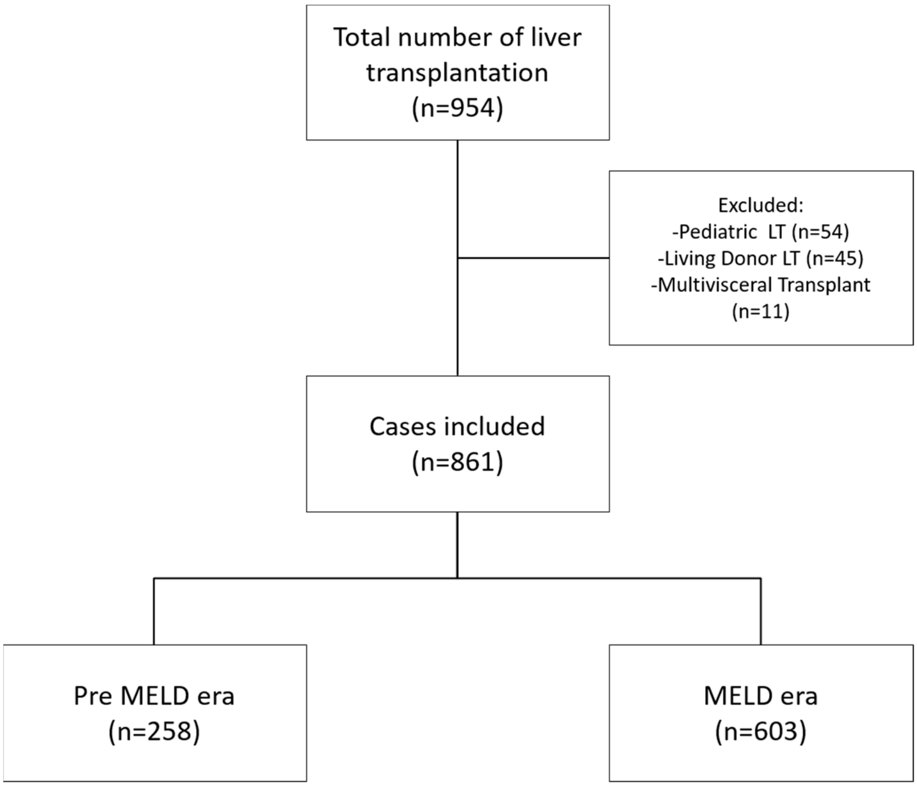

Figure S1. Patients included in the study.

Between 1 January 2005 and 31 December 2012, 954 liver transplantations were performed at the Department of Surgery at Charité University Hospital, Berlin, Campus Virchow and Campus Mitte. Among these 54 pediatric liver transplantations, 45 living donor liver transplantations and 11 combined organ transplantation other than liver-kidney transplantation. These patients were not included in the study as their allocation algorithm and their access to donor organs differ. This left 861 cases to be included in the analysis (some patients fulfilled multiple exclusion criteria).

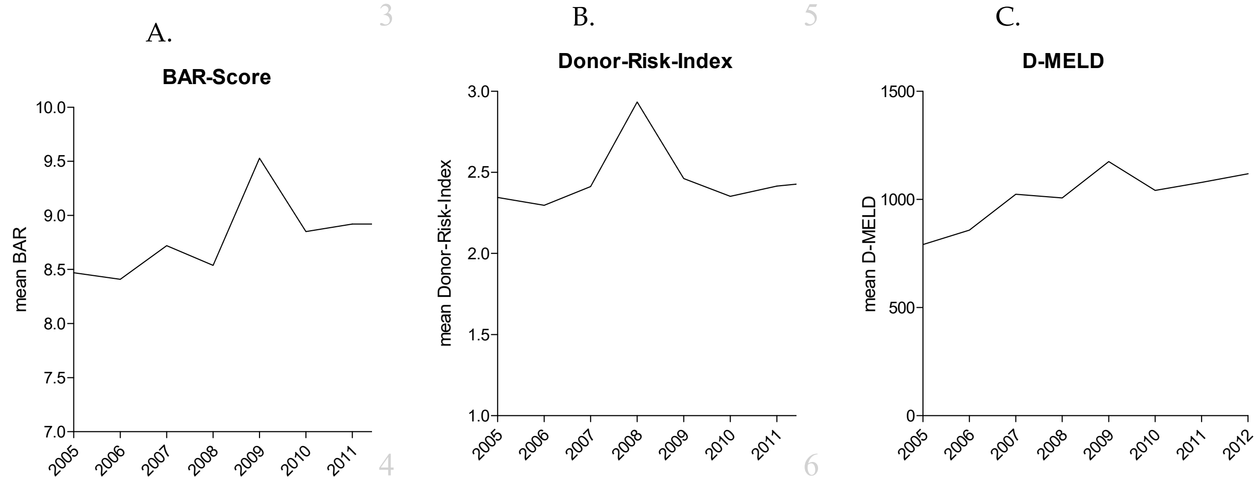

Figure 2. Development of established risk scores for liver transplantation after the implementation of MELD-based liver allocation.

The BAR-Score (A; including labMELD, recipient age, cold ischemia time, previous liver transplantation, life support before transplantation and donor age), the Donor-Risk-Index (B; including donor age, donor race, donor height, split graft, cold ischemia time, organ share, cause of

death and donation after cardiac death) and D-MELD (C; including labMELD and donor age) all represent well-known risk scores for survival after liver transplantation. Although significance could only be shown for D-MELD (Pearson's R = 0.168; $p < 0.001$, CI 0.101-0.233), they all show a trend to increasing values in the last years at our center. (BAR: Balance of Risk; MELD: Model for End-Stage Liver Disease)

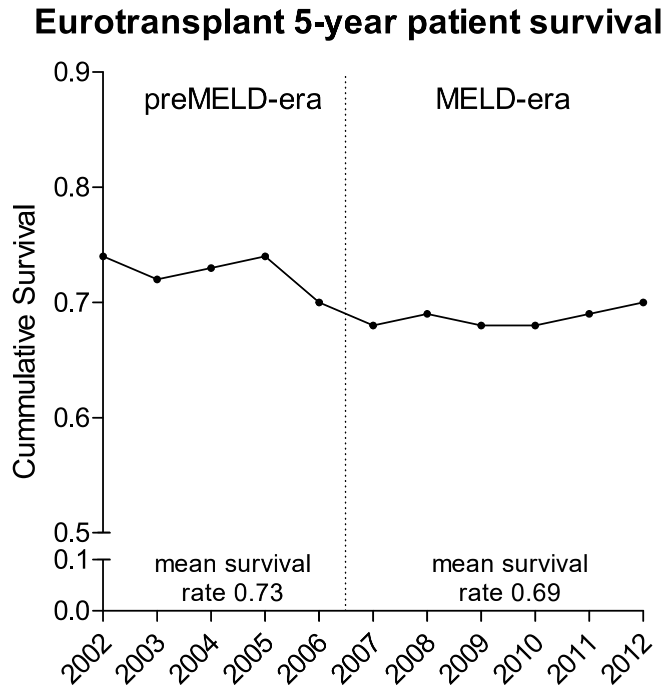

Figure S3. Development of 5-year patient survival from 2002 to 2012 in the whole Eurotransplant area.

Mean survival in the eras is calculated by weighing the numbers of liver transplantation in single years. Mean 5-year survival rate between 2002 and 2006 - before implementation of MELD-based liver allocation was 0.73 in the whole Eurotransplant area, but only 0.69 between 2007 and 2012 – after the implementation in Germany. During 2002 and 2012 59.2% to 67.0% of all liver transplantations in the Eurotransplant countries were performed in Germany.
